# Bone Mass Loss and Sarcopenia in Ecuadorian Patients

**DOI:** 10.1155/2020/1072675

**Published:** 2020-03-17

**Authors:** M. Intriago, G. Maldonado, R. Guerrero, O. D. Messina, C. Rios

**Affiliations:** ^1^Universidad Espíritu Santo, Km 2.5 Vía La Puntilla, Samborondón, Guayaquil, Ecuador; ^2^General Hospital, Pi y Margall 750, C1155AHD CABA, Buenos Aires, Argentina

## Abstract

**Objective:**

To study the association between osteoporosis and sarcopenia and determine the prevalence of osteosarcopenia in patients who attended a rheumatology center in Ecuador.

**Methods:**

A cross-sectional study was conducted in a population of patients who had a densitometric study. The diagnosis of sarcopenia was determined by the DXA standard gold test, screening, and conventional methods (bioimpedance, anthropometric measurements, SARC-F, muscle function, and gait test).

**Results:**

A total of 92 patients were studied. The median age was 66 ± 10, 90% females. Using the criteria of SMI, 65% had sarcopenia of which 9% had only sarcopenia and 56% had osteosarcopenia; 22% had only osteopenia/osteoporosis; and 13% none of these conditions. The prevalence of sarcopenia according to handgrip strength was 60%, gait speed 45%, and SARC-F score 40%. The prevalence of osteosarcopenia according to handgrip strength was 51%, gait speed 34%, and SARC-F score 32%. Osteoporosis was associated with a higher prevalence of sarcopenia using the criteria of SMI since 40% had sarcopenia in the normal DXA group, 64% in the osteopenia group, and 76% in the osteoporosis group (*p*=0.017). Of the women, 69% had sarcopenia compared to 33% of the men (*p*=0.034). The BMI was lower in the group with sarcopenia (25.1 ± 4.1 kg/m^2^) compared to the group without sarcopenia (29.4 ± 4.1 kg/m^2^, *p* < 0.001). Patients with osteosarcopenia and sarcopenia had lower BMI, handgrip strength, ASM, SMI, and total-body skeletal muscle mass than those with osteopenia/osteoporosis or normal patients.

**Conclusion:**

65% of the studied population had sarcopenia. It is clear that the prevalence of sarcopenia is higher in patients with greater loss of bone mass. Identifying pathways that affect both bone and muscle could facilitate the development of treatments that simultaneously improve osteoporosis and sarcopenia.

## 1. Introduction

The aging process is associated with concomitant loss of bone and muscle which increases the risk of falls and fractures and is associated with poor clinical outcomes and increases the use of resources. Osteoporotic fractures are a major cause of morbidity and mortality in elderly patients and generate costs that reach $25 billion [1]. Likewise, sarcopenia affects the mobility of patients and is related to a higher rate of disability, frailty, and hospitalizations, resulting in costs around $18 billion [2]. The term osteosarcopenia was recently proposed to describe the coexistence of both conditions in the same patient [3]. It has been shown that this combination represents an even greater risk of these results than any of the conditions alone [4].

Several theories have been proposed to explain the close relationship between osteoporosis and sarcopenia. The mechanostatic hypothesis describes the mechanical effects of muscle load on bone function, which provides a direct stimulus that promotes osteogenesis [5]. On the contrary, during the last decade, it has been shown that the interaction between bone and muscle is not only due to its contiguity but also by different signals that regulate its growth and adaptation. In this way, it is postulated that there is a paracrine and endocrine communication between both tissues through the action of different growth factors, hormones, and inflammatory mediators [6].

At present, there are few epidemiological studies about osteosarcopenia due to the recent use of this term. Fahimfar et al. [7] found a prevalence of osteosarcopenia of 34% in men and 34% in women. In a meta-analysis, the prevalence of this condition varied between 5 and 37% [8]. Likewise, Huo et al. [9] reported a prevalence of 40% in a cohort of patients with a history of falls. Regarding Latin America, Tramontano et al. [10] found a prevalence of sarcopenia of 18% in Peru, Gonzalez et al. [11] found a prevalence of 53% in Colombia, and Lera et al. [12] found a prevalence of 19% in Chile. In Ecuador, Maldonado and Ríos [13] determined a prevalence of sarcopenia of 66%; however, they did not study the relationship of this condition with osteoporosis.

Due to the frequent coexistence of sarcopenia-osteoporosis and its clinical implications, the objective of this study was to study the association between both pathologies and determine the prevalence of osteosarcopenia in patients who attended a rheumatology center in Ecuador.

## 2. Materials and Methods

A cross-sectional study was conducted in a population of patients who attended a rheumatology center in Guayaquil-Ecuador during the period of January 2017-2018. We included patients older than 50 years who had had a bone densitometry in the clinic and who were requested tests to measure muscle mass. Patients of younger age or who had some type of disability were excluded from the study. Prior to their participation, patients were informed of the purpose of the study, and informed consent was requested. Likewise, before the beginning of the study, the Institutional Review Board of the clinic reviewed the research protocol and authorized its completion.

Demographic data were obtained that included age, sex, and age of menopause in women. The following measurements were obtained: weight in kilograms, height in meters, handgrip strength using a hydraulic dynamometer, gait speed measured on a 4 meter distance, total-body skeletal muscle mass, and appendicular skeletal muscle mass (ASM) measured by dual energy X-ray absorptiometry (DXA). These tests were performed by a previously trained investigator. In addition, subjects filled out the SARC-F questionnaire in their native language (Spanish) after an explanation of it. The body mass index (BMI) was calculated with the patient's weight and height. Subjects were considered to have normal weight if their BMI was between 18.5 and 24.9 kg/m^2^, underweight if less than 18.5 kg/m^2^, overweight 25–29.9 kg/m^2^, and obese if it was 30 kg/m^2^ or more [14]. Likewise, we calculated the skeletal muscle mass index (SMI) with the ASM and height.

The cutoff for sarcopenia using SARC-F was equal to or greater than 4 [15]. This questionnaire is used for the screening of sarcopenia and has demonstrated a sensitivity of 29.5% and specificity of 98.1% [16]. The cutoff used for the other tests was based on the 2010 criteria of the European Working Group on Sarcopenia in Older People (EWGSOP) [17]. For handgrip strength, values <30 kg for men and <20 kg for women were considered sarcopenia. This test has demonstrated a sensitivity of 63% and specificity of 70% [18]. The cutoff for gait speed was ≤0.8 m/s for both sexes. Using DXA as the gold standard, the cutoff points for SMI were ≤7.23 kg/m^2^ in men and ≤5.67 kg/m^2^ in women.

Regarding bone mineral density, the results of the most recent DXA scan of the patient was obtained from the medical history and recorded. Following the criteria of the World Health Organization [19], patients with a *T* score of −1.0 or higher were considered normal, −1.0 and −2.5 osteopenia and −2.5 or lower osteoporosis. Osteosarcopenia was defined as the presence in the same subject of osteoporosis or osteopenia according to DXA and sarcopenia according to the criteria of SMI.

Data were analyzed using the program SPS v22. We used the Kolmogorov–Smirnov test to verify the normality of continuous variables. The description of the results was made with percentages, mean, and standard deviation. The ANOVA test was used to validate the differences in means between more than two groups and Student's *t*-test between two groups. For categorical variables, we used Chi-square. Statistical significance was less than 0.05.

## 3. Results

We studied 92 subjects with a mean age of 66 ± 10 years (minimum 50 years, maximum 89 years). The majority of them (90%) was women with a mean age of 65 ± 10 years, while the mean age of men was 67 ± 9 years. All women in the study were postmenopausal, and all men were over 50 years.

The mean weight was 63.2 ± 13 kg, height 1.5 ± 0.1 m, and BMI 26.7 ± 4 kg/m^2^. According to the BMI, 3% were found to be underweight, 27% ideal weight, 50% overweight, and 20% obese. According to the DXA, 22% were normal, 24% osteopenia, and 54% osteoporosis.

Using the criteria of SMI, 65% had sarcopenia of which 9% had only sarcopenia and 56% had osteosarcopenia, 22% had only osteopenia/osteoporosis, and 13% none of these conditions. The prevalence of sarcopenia according to handgrip strength was 60%, gait speed 45%, and SARC-F score 40%. The prevalence of osteosarcopenia according to handgrip strength was 51%, gait speed 34%, and SARC-F score 32%.

Osteoporosis was associated with a higher prevalence of sarcopenia using the criteria of SMI since 40% had sarcopenia in the normal DXA group, 64% in the osteopenia group, and 76% in the osteoporosis group (*p*=0.017). According to the gait speed, 50% of patients in the normal group had sarcopenia, 36% in the osteopenia group, and 46% in the osteoporosis group (*p*=0.644). According to handgrip strength, sarcopenia was found in 40% of normal, 59% of patients with osteopenia, and 69% of the osteoporosis group (*p*=0.076). According to the SARC-F screening questionnaire, 38% of the normal group, 50% of the osteopenia group, and 36% of the osteoporosis group had sarcopenia (*p*=0.936).

Table 1 shows the comparison of sarcopenia tests between groups with normal DXA, osteopenia, and osteoporosis. The mean BMI was 28.1 ± 3 kg/m^2^ in the normal group, 27.7 ± 4 kg/m^2^ in the osteopenia group, and 25.6 ± 5 kg/m^2^ in the osteoporosis group (*p*=0.067). The osteopenia group had lower gait speed, while the osteoporosis group had lower handgrip strength; however, these differences were not statistically significant. On the contrary, patients with osteoporosis had lower mean ASM and total-body skeletal muscle mass than patients with osteopenia and normal DXA (*p*=0.013, respectively). We did not find any significant difference in the SMI between the groups.

When analyzing the group of patients with and without sarcopenia according to the gold standard, we found that the ages in both groups were similar: 67.1 ± 10.4 years in patients with sarcopenia compared to 64.0 ± 9.1 years in those without sarcopenia (*p*=0.149). Of the women, 69% had sarcopenia compared to 33% of the men (*p*=0.034). The BMI was lower in the group with sarcopenia (25.1 ± 4.1 kg/m^2^) compared to the group without sarcopenia (29.4 ± 4.1 kg/m^2^, *p* < 0.001). Likewise, the prevalence of overweight/obesity was higher in patients without sarcopenia (88%) than in subjects with sarcopenia (60%, *p*=0.006).  Table 2 shows the mean *T* score according to the group. Patients with sarcopenia had lower *T* scores in all the measured areas.

Patients with osteosarcopenia and sarcopenia had lower BMI, handgrip strength, ASM, SMI, and total-body skeletal muscle mass than those with osteopenia/osteoporosis or normal patients.  Table 3 shows the characteristics of the patients according to the presence of osteosarcopenia or sarcopenia.

## 4. Discussion

The results of this study show that the prevalence of sarcopenia and osteosarcopenia in elderly patients is high and varies depending on the classification used for their diagnosis. According to the EWGSOP 2010 criteria, 65% of patients presented sarcopenia, most of them with osteosarcopenia. In the study by Scott et al. [20], the prevalence of sarcopenia alone was 7% and osteosarcopenia 8%, which is significantly lower than what was found in this study. This difference may be due to the fact that their cohort included only men, while in this study, the majority was women, who have shown to have a higher prevalence of muscle mass loss [9]. Another study showed a prevalence of osteosarcopenia of 27% and sarcopenia of 10% [4]. Something more similar to our study was seen in the study by Yoo et al. [21] where the reported prevalence of sarcopenia was 44% in women and 68% in men who had had a previous fracture. These differences in prevalence may be due to the different criteria used to define sarcopenia. In addition, the prevalence of overweight/obesity in this study was high, and obesity has been independently related to osteosarcopenia and sarcopenia. Huo et al. [9] showed that a high-fat mass is related to the presence of sarcopenia in men and women. The mechanism proposed to explain this relationship is the proinflammatory state and insulin resistance generated by central adiposity which is directly related to the loss of muscle mass [22].

It is estimated that the loss of muscle and bone mass begins at the end of the 20 years and accelerates in the 50s [23]. Lang et al. [24] predicted that muscle mass decreases by approximately 40% between the ages of 20 and 60 with an average of approximately 1% per year. Although not significant, patients with sarcopenia in this study were older than patients without sarcopenia. Overall, the mean age of the study patients was 66 years, which is lower than the average age of other studies [9, 25].

We found a significant association between sarcopenia and osteoporosis since most of the patients in the osteoporosis group had sarcopenia, and the majority of sarcopenia cases presented as osteosarcopenia. Di Monaco et al. [26] also found a significant association between sarcopenia and osteoporosis with a prevalence of sarcopenia of 58% and osteoporosis of 74% in a population of women with hip fractures. In addition, these authors calculated that the odds ratio of osteoporosis in a woman with sarcopenia is 1.8 [26].

In the meta-analysis by Nielsen et al. [8], the prevalence of sarcopenia in patients with low energy fracture was 46%, with a relative risk of fracture of 1.37 in patients with sarcopenia. In addition, they found a difference of 0.07 g/cm^2^ in bone mineral density and −0.34 in the *T* score in patients with sarcopenia compared to patients without sarcopenia [8]. This relationship is similar to that found in this study, where the *T* score of patients with sarcopenia was significantly lower than that of patients without sarcopenia. On the contrary, Hong et al. [27] determined the prevalence of sarcopenia according to the type of fragility fracture and found sarcopenia in 25% of women with wrist fractures, 21% in women with ankle fractures, 34% in women with vertebral fractures, and 42% in women with hip fractures, compared to 18% in women without any fractures. Likewise, similar to our study, they found a correlation between DXA values and the skeletal muscle mass index.

In this study, we also found a significant difference in the total-body skeletal muscle mass and ASM between the groups with osteoporosis, osteopenia, and normal. In the same way, Verschueren et al. [28] reported a positive correlation between appendicular lean mass and appendicular skeletal muscle mass with DXA. These authors found that patients with a relative appendicular skeletal muscle mass <7.26 kg/m^2^ had a significantly lower DXA than those with muscle mass above this value. In addition, these patients were more likely to have osteoporosis.

As has been shown in several studies, greater muscle mass protects against osteoporosis and also an increase in bone density decreases the risk of sarcopenia [29]. Mechanical loading is a key mechanism that links both tissues, thereby enhancing the role of physical activity in maintaining musculoskeletal health. Osteocytes detect the mechanical stress of muscle contraction and stimulate osteoblasts to increase bone mineral density and resistance at the site that is under the greatest pressure [30].

It has also been shown that sarcopenia and osteoporosis share other common mechanisms that include reduction of anabolic hormones, increase in inflammatory cytokines, and release of myokines and osteokines [31]. Among the molecules studied, the role of insulin-like growth factor, interleukin 6, interleukin 15, myostatin, osteoactivin, osteocalcin, and prostaglandin E2 has been highlighted [32]. Vitamin D also plays an important role as demonstrated by the study by Tanaka et al. [33] in which vitamin D induced the expression of myogenin and osteoglycin, promoting myogenesis and osteoblastogenesis. Finally, since both myocytes and bone cells derive from the same embryonic origin, they are affected by similar genetic factors. Karasaki and Kiel [34] estimated that the risk factors that affect osteoporosis and sarcopenia are hereditary at approximately 60 to 70%.

The study has several limitations. First, the sample size is small. Also, most of the population was women which could skew the study. We did not include the main clinical diagnosis or treatments used by patients. This is of concern as the study was conducted in a rheumatology clinic where there could be pathologies and medications that might contribute by themselves to bone and muscle mass loss. Finally, as it is a cross-sectional study, it is difficult to establish causal relationships between osteoporosis and sarcopenia.

## 5. Conclusion

The prevalence of sarcopenia and osteosarcopenia was high in a rheumatology clinic. It is clear that the prevalence of sarcopenia is higher in patients with greater bone loss. Because these two conditions are commonly seen in the elderly population, protocols that include their management are required. The identification of pathways that affect both bone and muscle could facilitate the development of treatments that simultaneously improve osteoporosis and sarcopenia.

## Figures and Tables

**Table 1:** Sarcopenia tests according to DXA diagnoses.

	Normal (*n* = 20)	Osteopenia (*n* = 22)	Osteoporosis (*n* = 50)	*p*
Gait speed (m/s)	0.8 ± 0.2	0.7 ± 0.4	0.9 ± 1.2	0.640
Handgrip strength (kg)	20.2 ± 6.7	23.2 ± 16.2	18.2 ± 7.3	0.156
SARC-F	3.3 ± 2.1	3.3 ± 2.3	2.8 ± 2.1	0.747
ASM (kg)	17.9 ± 3.5	16.5 ± 3.9	15.3 ± 3.2 kg	0.013
Total-body skeletal muscle mass (kg)	23.9 ± 4.7	21.9 ± 5.3	20.3 ± 4.7	0.013
SMI (kg/m^2^)	7.3 ± 0.9	6.7 ± 1.1	6.6 ± 1.1	0.072

**Table 2:** Mean *T* score in patients with and without sarcopenia using SMI criteria.

	With sarcopenia (*n* = 60)	Without sarcopenia (*n* = 32)	*p*
Left hip	−1.3 ± 0.6	−0.7 ± 0.9	0.003
Right hip	−1.6 ± 0.7	−0.9 ± 1.2	0.001
*L*1	−1.9 ± 1.1	−1.3 ± 1.1	0.044
*L*2	−1.9 ± 1.2	−1.4 ± 1.1	0.010
*L*3	−2.2 ± 1.0	−1.3 ± 1.2	0.004
*L*4	−2.1 ± 1.1	−1.2 ± 1.1	0.003

**Table 3:** Characteristics of patients according to sarcopenia and osteoporosis diagnoses.

		Normal (*n* = 12)	Osteopenia-osteoporosis (*n* = 20)	Osteosarcopenia (*n* = 52)	Sarcopenia (*n* = 8)	*p*
Age (years)		63 ± 8	64 ± 9	67 ± 11	66 ± 4	0.502
Female (%)		75	85	94	100	0.134
BMI (kg/m^2^)		29.6 ± 2	29.3 ± 4	25.2 ± 4	25.6 ± 2	<0.001
Overweight/obesity (%)	92	85	60	63	0.054
SARC-F	2.0 ± 1	2.6 ± 2	3.1 ± 2	4.2 ± 2	0.510
Gait speed (m/s)	0.9 ± 0.3	0.9 ± 0.5	0.9 ± 1.2	0.7 ± 0.1	0.893
Handgrip strength (kg)	23.9 ± 4	25.7 ± 7	17.4 ± 5	14.5 ± 5	0.002
ASM (kg)	20.2 ± 2	19.4 ± 3	14.2 ± 2	14.7 ± 2	<0.001
SMI (kg/m^2^)	7.9 ± 0.5	7.9 ± 0.9	6.1 ± 0.7	6.2 ± 0.4	<0.001
Total-body skeletal muscle mass (kg)	26.7 ± 3	25.7 ± 4	18.9 ± 2	19.6 ± 2	<0.001

## Data Availability

The authors confirm that the data supporting the findings of this study are available within the article.

## References

[1] Dempster D. W. (2011). Osteoporosis and the burden of osteoporosis-related fractures. *The American Journal of Managed Care*.

[2] Janssen I., Shepard D. S., Katzmarzyk P. T., Roubenoff R. (2004). The healthcare costs of sarcopenia in the United States. *Journal of the American Geriatrics Society*.

[3] Binkley N., Buehring B. (2009). Beyond FRAX: it’s time to consider “Sarco-Osteopenia”. *Journal of Clinical Densitometry*.

[4] Yoo J.-I., Kim H., Ha Y.-C., Kwon H.-B., Koo K.-H. (2018). Osteosarcopenia in patients with hip fracture is related with high mortality. *Journal of Korean Medical Science*.

[5] Frost H. M. (2003). Bone’s mechanostat: a 2003 update. *The Anatomical Record*.

[6] Girgis C. M., Mokbel N., Digirolamo D. J. (2014). Therapies for musculoskeletal disease: can we treat two birds with one stone?. *Current Osteoporosis Reports*.

[7] Fahimfar N., Zahedi Tajrishi F., Gharibzadeh S. (2019). Prevalence of osteosarcopenia and its association with cardiovascular risk factors in Iranian older people: bushehr elderly health (BEH) program. *Calcified Tissue International*.

[8] Nielsen B. R., Abdulla J., Andersen H. E., Schwarz P., Suetta C. (2018). Sarcopenia and osteoporosis in older people: a systematic review and meta-analysis. *European Geriatric Medicine*.

[9] Huo Y. R., Suriyaarachchi P., Gomez F. (2015). Phenotype of osteosarcopenia in older individuals with a history of falling. *Journal of the American Medical Directors Association*.

[10] Tramontano A., Veronese N., Sergi G. (2017). Prevalence of sarcopenia and associated factors in the healthy older adults of the Peruvian Andes. *Archives of Gerontology and Geriatrics*.

[11] Gonzalez-Gonzalez D., Lopez-Salazar A., Gonzalez-Correa C., Curcio-Borrero C. (2019). Prevalence of sarcopenia among community- dwelling, young elderly people living in Manizales, Colombia. *Journal of Physics Conference Series*.

[12] Lera L., Albala C., Sánchez H. (2017). Prevalence of sarcopenia in community-dwelling Chilean elders according to an adapted version of the European working group on sarcopenia in older people (EWGSOP) criteria. *The Journal of Frailty & Aging*.

[13] Maldonado G., Ríos C. (2013). Prevalence of sarcopenia in Ecuadorian population based on screening and diagnostic tools. *Annals of the Rheumatic Diseases*.

[14] Ulijaszek Stanley J. (2003). Obesity: preventing and managing the global epidemic. report of a WHO consultation. WHO technical report series 894. Pp. 252. (world health organization, Geneva, 2000.) SFr 56.00, ISBN 92-4-120894-5, paperback. *Journal of Biosocial Science*.

[15] Malmstrom T. K., Morley J. E. (2013). SARC-F: a simple questionnaire to rapidly diagnose sarcopenia. *Journal of the American Medical Directors Association*.

[16] Yang M., Hu X., Xie L. (2019). Comparing mini sarcopenia risk assessment with SARC-F for screening sarcopenia in community-dwelling older adults. *Journal of the American Medical Directors Association*.

[17] Cruz-Jentoft A. J., Baeyens J. P., Bauer J. M. (2010). Sarcopenia: European consensus on definition and diagnosis: report of the European working group on sarcopenia in older people. *Age and Ageing*.

[18] Falson M., Harris-Love Michael O. (2017). Sarcopenia and the new ICD-10-CM Code: screening, staging, and diagnosis considerations. *Federal Practitioner*.

[19] WHO (2000). *WHO Scientific Group on the Prevention and Management of Osteoporosis: Prevention and Management of Osteoporosis*.

[20] Scott D., Seibel M., Cumming R. (2019). Does combined osteopenia/osteoporosis and sarcopenia confer greater risk of falls and fracture than either condition alone in older men? the concord health and ageing in men project. *The Journals of Gerontology: Series A*.

[21] Yoo J.-I., Ha Y.-C., Kwon H.-B., Lee Y.-K., Koo K.-H., Yoo M.-J. (2016). High prevalence of sarcopenia in Korean patients after hip fracture: a case-control study. *Journal of Korean Medical Science*.

[22] Gabat J. A. L., Faltado A. L., Sedurante M. B., Tee M. L. (2018). Association of obesity and sarcopenia among adult filipinos. *Osteoporosis and Sarcopenia*.

[23] Ormsbee M. J., Prado C. M., Ilich J. Z. (2014). Osteosarcopenic obesity: the role of bone, muscle, and fat on health. *Journal of Cachexia, Sarcopenia and Muscle*.

[24] Lang T., Streeper T., Cawthon P., Baldwin K., Taaffe D. R., Harris T. B. (2010). Sarcopenia: etiology, clinical consequences, intervention, and assessment. *Osteoporosis International*.

[25] Frisoli A., Chaves P., Inghan S., Carvalho A. (2018). Osteosarcopenia has stronger association with impaired physical function than sarcopenia only. *Innovation in Aging*.

[26] Di Monaco M., Vallero F., Di Monaco R., Tappero R. (2011). Prevalence of sarcopenia and its association with osteoporosis in 313 older women following a hip fracture. *Archives of Gerontology and Geriatrics*.

[27] Hong W., Cheng Q., Zhu X. (2015). Prevalence of sarcopenia and its relationship with sites of fragility fractures in elderly Chinese men and women. *PLoS One*.

[28] Verschueren S., Gielen E., O’Neill T. W. (2013). Sarcopenia and its relationship with bone mineral density in middle-aged and elderly European men. *Osteoporosis International*.

[29] Da Silva A. P., Matos A., Ribeiro R. (2017). Sarcopenia and osteoporosis in Portuguese centenarians. *European Journal of Clinical Nutrition*.

[30] Rochefort G. Y., Pallu S., Benhamou C. L. (2010). Osteocyte: the unrecognized side of bone tissue. *Osteoporosis International*.

[31] Isaacson J., Brotto M. (2014). Physiology of mechanotransduction: how do muscle and bone “talk” to one another?. *Clinical Reviews in Bone and Mineral Metabolism*.

[32] Tagliaferri C., Wittrant Y., Davicco M.-J., Walrand S., Coxam V. (2015). Muscle and bone, two interconnected tissues. *Ageing Research Reviews*.

[33] Tanaka K.-I., Kanazawa I., Yamaguchi T., Yano S., Kaji H., Sugimoto T. (2014). Active vitamin D possesses beneficial effects on the interaction between muscle and bone. *Biochemical and Biophysical Research Communications*.

[34] Karasik D., Kiel D. P. (2008). Genetics of the musculoskeletal system: a pleiotropic approach. *Journal of Bone and Mineral Research*.

